# Prevention of spinal fusion post-operative wound infections in pediatric patients with scoliosis: a quality improvement initiative

**DOI:** 10.1007/s43390-020-00274-3

**Published:** 2021-01-13

**Authors:** Elizabeth Partridge, Dean Blumberg, Rolando F. Roberto

**Affiliations:** 1 University of California Davis Medical Center, Pediatric Infectious Disease, Sacramento, CA USA; 2 University of California Davis Medical Center, Orthopedics, Sacramento, CA USA; 3 Shriners Hospitals for Children – Northern California, Orthopedics, Sacramento, CA USA

**Keywords:** Spinal fusion, Scoliosis, Post-operative wound infections, Surgical site infection, Surgical prophylaxis, Neuromuscular scoliosis

## Abstract

**Purpose:**

Post-operative wound infections increase patient morbidity and mortality as well as the length of hospital stay, with a profound personal and institutional cost. The aim of this study was to decrease post-operative infections through development of a surgical antibiotic prophylaxis policy based on institution-specific risk factors and microbiology data.

**Methods:**

We conducted a retrospective review of deep wound infections at our institution over a 5-year period (2014–2018). 399 spinal fusion procedures were performed with a 2.5% post-operative infection rate. Patients with neuromuscular scoliosis were six times more likely to develop deep wound infections (7.6%) compared to patients with congenital and idiopathic scoliosis (combined rate of 1.25%). The microbiology data revealed that polymicrobial, extended spectrum beta-lactamase (ESBL) gram negative organisms predominated in patients with neuromuscular scoliosis. Based on these findings, we implemented an evidence-based quality improvement intervention: all patients with neuromuscular scoliosis undergoing spinal fusion were given a single 15 mg/kg dose of amikacin, in addition to our standard practice of perioperative cefazolin plus vancomycin with intra-operative betadine wash and vancomycin powder application. This intervention was put into practice in January 2019.

**Results:**

Since the implementation of our quality improvement initiative, the overall post-operative infection rate decreased to 1.1% (2 infections in 176 cases). Ninety-eight percent of the 43 neuromuscular scoliosis patients who underwent spinal fusion in the post-intervention time frame have remained infection free.

**Conclusion:**

Examination of post-operative infection and microbiology data at the institution level can guide the development of institution specific, evidence-based quality improvement initiatives that reduce post-operative wound infections.

## Background

Post-operative wound infections increase patient morbidity and mortality as well as the length of hospital stay, with a profound personal and institutional cost. The CDC healthcare-associated infection (HAI) prevalence survey estimated 110,800 surgical site infections (SSIs) associated with inpatient surgeries in 2015. Between 2015 and 2018, there was a 5% decrease in the standardized infection ratio (SIR) related to all National Health and Safety Network (NHSN) operative procedure categories. Several infection control practices have contributed to this reduction in SSIs including improved operating room ventilation, sterilization methods, surgical technique and availability of antimicrobial prophylaxis. Despite these advances, SSI is associated with a morality rate of 3% and remains the most costly type of HAI with an estimated additional 1 million in-patient days and $3.3 billion annual cost [1–3].

The incidence of SSIs in pediatric patients undergoing spinal fusion with and without instrumentation ranges from 2.5 to 5.2%. Cefazolin (single dose, timed to achieve bactericidal concentration in the serum and tissues at the time of incision) is recommended for SSI prophylaxis in spinal procedures given its broad spectrum of activity (e.g. against methicillin susceptible *Staphylococcus aureus* and gram-negative bacteria such as *E. coli*) and excellent penetration into tissue and disc space. Clindamycin and vancomycin are alternative agents in patients with beta-lactam allergy. In patients with known MRSA colonization, vancomycin should be used as surgical prophylaxis in addition to a topical agent for intranasal decolonization. Importantly, for procedures in which gram-negative pathogens are likely, or surveillance data indicate that gram-negative organisms are a cause of SSIs, practitioners may consider combining clindamycin or vancomycin with another agent (i.e. cefazolin, aztreonam, gentamicin or single-dose fluroquinolone) [4, 5]. Thus, modification of standard prophylaxis to account for local SSI epidemiology, as done in our quality improvement initiative, follows the ASHP (American Society of Health-System Pharmacists) surgical prophylaxis guidelines.

## Methods

In addition to routine, active, patient-based, prospective surveillance for SSI surveillance using NHSN criteria, we conducted a retrospective review of deep wound infection data over a 5-year period (2014–2018) at our institution. Deep incisional SSIs were those occurring within 90 days after spinal fusion (NHSN operative procedure category FUSN) that involved deep soft tissues of the incision (i.e. fascia and muscle layers) with either purulence or a positive culture (or non-culture based microbiologic testing method) from deep soft tissues in patients with at least one of the following clinical criteria: fever (> 38 °C); localized pain or tenderness or evidence of deep abscess [6, 7]. Per hospital protocol, all surgical patients received pre-operative MRSA nasal screening (by culture) and if positive, underwent a decolonization protocol with BID nasal mupirocin and daily chlorohexidine baths for 5 days prior to surgery. All patients, regardless of MRSA screening results, received routine SSI prophylaxis prior to incision and for 24hrs post-op with vancomycin plus cefazolin. Prior to closure, all patients received betadine wash and vancomycin powder per hospital protocol.

## Results

**Fig. 1:**
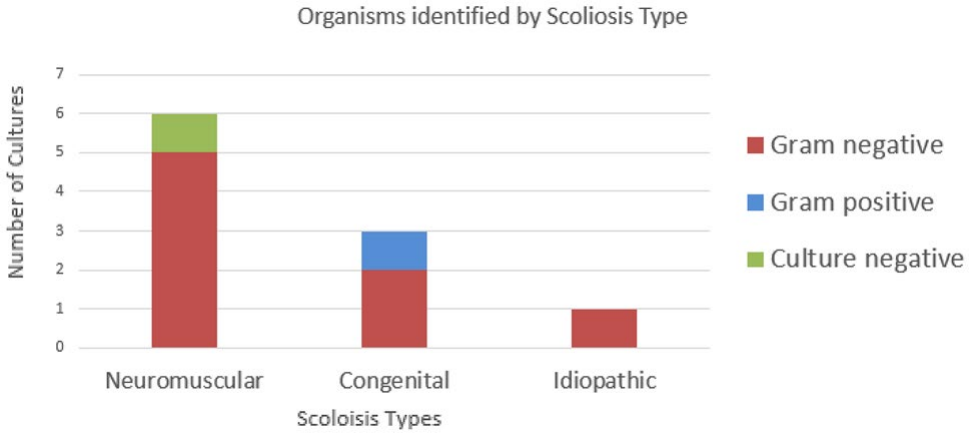
This figure summarizes the number of cultures (y-axis) by scoliosis type (x-axis) and culture result (i.e. gram-negative organism isolated in red, gram-positive organism in blue or no organisms isolated in green)

Retrospective review revealed 399 spinal fusion procedures performed from 2014–2018 with an overall post-op infection rate of 2.5%. The majority of spinal fusion procedures were performed on patients with either congenital or idiopathic scoliosis (320). While only 20% of patients that underwent spinal fusion had neuromuscular scoliosis, they were six times more likely to develop deep wound infections (7.6%) compared to patients with congenital and idiopathic scoliosis (combined rate of 1.25%) (Table 1). Microbiology data revealed that polymicrobial, extended-spectrum beta-lactamase (ESBL) gram-negative organisms were the predominant organisms identified from SSIs of patients with underlying neuromuscular scoliosis (Fig. 1). While susceptibility to gentamicin varied, all gram-negative isolates were susceptible to amikacin (Table 2).

**Table 1:** The numbers of surgeries, infections and rate of infection by scoliosis type (idiopathic and congenital vs neuromuscular) and by time period (2014–2018 vs. 2019–2020)

Time period	Scoliosis type			
	Idiopathic and congenital		Neuromuscular	
	2014–2018	2019–2020^a^	2014–2018	2019–2020^a^
# Surgeries	320	176	79	43
# Infections	4	1	6	1
Infection rate	1.25	0.5	7.6	2.3

^a^Data are through September 2020

**Table 2:** Organisms and susceptibilities of deep SSIs after spinal fusion by scoliosis type and year

Scoliosis type	Year	Organism	Susceptibilities
Idiopathic	2014	*Enterobacter*	S: amikacin, gentamicin
Congenital	2014	MSSA	
		ESBL *E. coli*	S: amikacin, gentamicin
	2015	*Enterobacter*	S: amikacin
Neuromuscular	2016	ESBL *Klebsiella*	S: amikacin, gentamicin
		ESBL *E. coli*	S: amikacin; R: gentamicin
	2017	ESBL *Klebsiella*	S: amikacin, gentamicin
		ESBL *E. coli*	S: amikacin; R: gentamicin
		ESBL *E. coli*	S: amikacin; R: gentamicin
	2018	ESBL *Pseudomonas*	S: amikacin, gentamicin
		*B. fragilis*	
		*E. coli*	S: amikacin; R: gentamicin
		ESBL *Enterobacter*	S: amikacin, gentamicin

### Intervention

Based on risk factors for post-operative infection as well as microbiology and susceptibility data, we instituted an evidence-based quality improvement intervention in which all patients with neuromuscular scoliosis undergoing spinal fusion were given a single 15 mg/kg dose of amikacin, in addition our standard practice of perioperative cefazolin plus vancomycin and intra-operative betadine wash and vancomycin powder application. This intervention was put into practice in January 2019. Of the 176 cases of a spinal fusion performed since the institution of our intervention, 2 cases of post-op infection were seen (1.1%), one in a patient with idiopathic scoliosis and the second in a patient with neuromuscular scoliosis. One SSI has occurred in the 43 neuromuscular scoliosis patients who underwent spinal fusion since the implementation of our quality improvement initiative (*p* = 0.4212 by fisher’s exact test) (Table 1). Further review of post-intervention infection rates, microbial and susceptibility data is on-going.

## Discussion

Our institutional rate of spinal fusion SSIs for the study period 2014–2018 is on the low end of national trends. Closer examination of post-operative infection and microbiology data beyond routine surveillance as mandated by the NHSN identified an opportunity for further reduction in post-operative wound infections within a sub-population of patients undergoing spinal fusion procedures at our institution. This resulted in a clinical practice change with the prospect for significant improvement in patient clinical outcomes and institutional cost. Since implementing this quality improvement project, only one SSI has developed in neuromuscular patients undergoing spinal fusion at our institution. Although our limited follow-up period has not revealed a statistically significant decrease in SSIs, the preliminary data are promising. Importantly, this QI initiative is differentiated from a research study in that no hypothesis was introduced and investigated. Therefore, no treatment recommendations can be made for institutions other than our own. Further limitations include the post-intervention cohort size and duration of the observation period. The study cohorts in the pre- and post-intervention periods were not compared regarding demographics, etiology, severity of the curves and procedures applied (length of procedure, extension of the fusion, osteotomies etc.); these comparisons are beyond the scope of this quality improvement project but could be considered in a follow-up study. Also, the unblinded nature of this project could result in provider behavior modifications other than our intervention, that impact patient outcome. These limitations may be overcome, and our findings confirmed by a further study using a more rigorous methodology (i.e. Randomized Controlled Trial).

In national clinical practice guidelines for antimicrobial prophylaxis in surgery, consideration is given to the addition of gentamicin to perioperative antibiotics if surveillance data implicate gram-negative organisms in SSIs. While several of our SSI isolates were resistant to gentamicin, all of them were susceptible to amikacin. Like gentamicin, amikacin is an aminoglycoside that is not typically given as monotherapy for post-operative wound infections thereby reducing the likelihood of resistance. While there is potential for renal toxicity, administration of a single dose is unlikely to cause lasting renal impact. At the time of this writing, we have 21 months of post-intervention data. Additional follow-up is not only more likely to yield statistically significant differences but will also provide further safety data detecting potential side effects such as nephrotoxicity associated with amikacin administration. Monitoring of post-intervention infection rates, microbial and susceptibility data is on-going. However, we believe the data in its current state are interesting and impactful enough to warrant sharing so that others may consider similar interventions. Considering the wide geographical and hospital variation in gram-negative susceptibilities, local surveillance data is a powerful tool for clinicians to further optimize SSI prevention in their own institutions.

## Conclusion

Examination of SSI surveillance data with specific attention to local microbiologic trends, can lead to evidence-based, institution-specific improvement initiatives that can reduce the personal and organizational cost of post-operative infections. We encourage each hospital and/or hospital system to implement quality improvement initiatives like this one, to optimize antibiotic prophylaxis and reduce their institutional SSI rates.

## Data Availability

via NSHN.
