# Correction to: Prevention of spinal fusion post-operative wound infections in pediatric patients with scoliosis: a quality improvement initiative

**DOI:** 10.1007/s43390-021-00299-2

**Published:** 2021-02-10

**Authors:** Elizabeth Partridge, Dean Blumberg, Rolando F. Roberto

**Affiliations:** 1 University of California Davis Medical Center, Pediatric Infectious Disease, Sacramento, CA USA; 2 University of California Davis Medical Center, Orthopedics, Sacramento, CA USA; 3 Shriners Hospitals for Children, Northern California, Orthopedics, Sacramento, CA USA

## Correction to: Spine Deformity 10.1007/s43390-020-00274-3

The original version of this article unfortunately contained a mistake. The following affiliation of all three authors had not been included and was added retrospectively: Shriners Hospitals for Children, Northern California, Orthopedics, Sacramento, CA, USA.

The original article has been corrected.

